# Pathways to care and preferences for improving tuberculosis services among tuberculosis patients in Zambia: A discrete choice experiment

**DOI:** 10.1371/journal.pone.0252095

**Published:** 2021-08-31

**Authors:** Andrew D. Kerkhoff, Mary Kagujje, Sarah Nyangu, Kondwelani Mateyo, Nsala Sanjase, Lophina Chilukutu, Ingrid Eshun-Wilson, Elvin H. Geng, Diane V. Havlir, Monde Muyoyeta

**Affiliations:** 1 Division of HIV, Infectious Diseases and Global Medicine Zuckerberg San Francisco General Hospital and Trauma Center University of California, San Francisco, California, United States of America; 2 Centre for Infectious Disease Research in Zambia, Lusaka, Zambia; 3 University Teaching Hospital, Department of Internal Medicine, Lusaka, Zambia; 4 Division of Infectious Diseases, Washington University School of Medicine, St. Louis, Missouri, United States of America; The University of Queensland, AUSTRALIA

## Abstract

**Background:**

Delays in the diagnosis of tuberculosis (TB) contribute to a substantial proportion of TB-related mortality, especially among people living with HIV (PLHIV). We sought to characterize the diagnostic journey for HIV-positive and HIV-negative patients with a new TB diagnosis in Zambia, to understand drivers of delay, and characterize their preferences for service characteristics to inform improvements in TB services.

**Methods:**

We assessed consecutive adults with newly microbiologically-confirmed TB at two public health treatment facilities in Lusaka, Zambia. We administered a survey to document critical intervals in the TB care pathway (time to initial care-seeking, diagnosis and treatment initiation), identify bottlenecks and their reasons. We quantified patient preferences for a range of characteristics of health services using a discrete choice experiment (DCE) that assessed 7 attributes (distance, wait times, hours of operation, confidentiality, sex of provider, testing incentive, TB test speed and notification method).

**Results:**

Among 401 patients enrolled (median age of 34 years, 68.7% male, 46.6% HIV-positive), 60.9% and 39.1% were from a first-level and tertiary hospital, respectively. The median time from symptom onset to receipt of TB treatment was 5.0 weeks (IQR: 3.6–8.0) and was longer among HIV-positive patients seeking care at a tertiary hospital than HIV-negative patients (6.4 vs. 4.9 weeks, p = 0.002). The time from symptom onset to initial presentation for evaluation accounted for the majority of time until treatment initiation (median 3.0 weeks, IQR: 1.0–5.0)–an important minority of 11.0% of patients delayed care-seeking ≥8 weeks.

The DCE found that patients strongly preferred same-day TB test results (relative importance, 37.2%), facilities close to home (18.0%), and facilities with short wait times (16.9%). Patients were willing to travel to a facility up to 7.6 kilometers further away in order to access same-day TB test results. Preferences for improving current TB services did not differ according to HIV status.

**Conclusions:**

Prolonged intervals from TB symptom onset to treatment initiation were common, especially among PLHIV, and were driven by delayed health-seeking. Addressing known barriers to timely diagnosis and incorporating patients’ preferences into TB services, including same-day TB test results, may facilitate earlier TB care engagement in high burden settings.

## Introduction

Tuberculosis (TB) killed an estimated 1.4 million individuals in 2019 and it remains the leading cause of death among people living with HIV (PLHIV) [1]. Delayed and missed TB diagnoses contribute to poor outcomes among predominantly socioeconomically vulnerable individuals, including prolonged suffering and mortality [2–4], as well as ongoing community transmission [5]. Therefore, continued progress in the fight against TB cannot be achieved without finding the missing persons with TB and identifying them sooner [3,5,6].

For individuals with undiagnosed TB, the pathway to care engagement may be long and complex as they often face numerous barriers to seeking care for their illness and accessing TB services [7–9]. These may include (among others): lack of knowledge about TB and its consequences, TB- and HIV-related stigma concerns, cultural and gender norms related to healthcare seeking, poverty and the direct and indirect costs of healthcare seeking, as well as perceptions that facilities do not meet their needs, including inflexible hours, long waiting times, judgmental or rude staff, and privacy and confidentiality concerns [7–10]. In order to design interventions that may improve health-seeking behavior for TB, it is important to understand contextually-specific barriers faced by individuals engaging in TB services, and also what intervention components would be the most preferred and acceptable. Discrete choice experiments (DCEs) are a quantitative method that quantifies patients’ preferences and allow trade-offs between program or intervention components to be evaluated [11,12]. However, to date, no study has undertaken a DCE among TB patients in order understand their preferences for improving TB care engagement.

TB is a leading cause of mortality in Zambia, especially among PLHIV who account for more than 60% of the annual 15,400 TB-related deaths [1,13]. We have previously identified that the largest gap in Zambia’s national TB care cascade (~73% of missed cases) is due to a lack of health-seeking and/or lack of access to TB diagnostic testing [14]. Yet, patient care journeys to TB diagnosis and treatment initiation for individuals in Zambia, including how they may differ according to HIV status, are not well defined. Furthermore, patients’ preferences for improving TB services are poorly understood. Thus, we sought to characterize the critical intervals in the pathways to care among newly diagnosed TB patients in Zambia, in order to identify potential bottlenecks, barriers they faced and understand whether these differed according to HIV status. We also undertook a DCE in order to determine patients’ preferences for improving TB services that could accelerate care engagement.

## Methods

### Setting and participants

This prospective cross-sectional study was undertaken at two public health facilities in Lusaka, Zambia from September 2019 to January 2020. Kanyama TB clinic is a busy outpatient TB clinic based at a first-level hospital that serves as the primary health facility for a large densely populated peri-urban township [15]. University Teaching Hospital (UTH) is a large tertiary referral hospital that serves all of Lusaka and has more than 1,600 beds.

Eligible participants were those aged ≥18 years, had microbiologically-confirmed TB, and had started on anti-TB therapy in the last 2 weeks (to reduce recall bias). Patients classified as a retreatment TB case (e.g., default, failure, lost-to-follow-up) were excluded due to potentially non-representative health-seeking and care experiences.

### Ethics, consent and permissions

All participants provided written informed consent in their preferred language (Bemba, Nyanja, or English). The study was approved by the University of Zambia Biomedical Research Ethics Committee (#010-014-19) and the institutional review board of the University of California, San Francisco (#18–26028).

### Procedures and survey

Participants completed a structured survey followed by a DCE. Surveys and DCEs were administered to participants using an electronic touchscreen tablet with the help of a trained research assistant. Surveys captured socio-demographic details and medical history (S1 Appendix). To understand patient pathways to care, surveys asked participants about their TB symptoms, timing of symptom onset, all care-seeking actions undertaken after symptom onset until TB diagnosis (and relative timing), as well as barriers and facilitators to seeking care for their TB illness and accessing TB care services. Additional details related to TB diagnosis and treatment history were extracted from facility-specific TB registers.

### DCE attribute selection and design

A DCE was administered to understand which potential features to improve TB services were most preferred by TB patients and could potentially improve health-seeking behaviors and result in earlier TB care engagement. TB health facility attributes and levels were informed by a literature review and discussions with key stakeholders [11]. The DCE design featured seven different TB service attributes that differed according to two to three levels (Table 1).

**Table 1:** Discrete choice experiment attributes and attribute levels to evaluate patient preferences for improving tuberculosis services in Zambia.

Attributes	Levels		
**1. Distance to facility (from home)**	2 kilometers	6 kilometers	10 kilometers
**2. Confidentiality**	A place where no one knows who I am	A place where I may be known or recognized	
**3. Hours of facility operation**	Normal weekday hours	Normal weekday hours + extended early morning or evening hours	Normal weekday hours + open Saturdays
**4. Sex of provider**	The healthcare provider is the same sex as I am	The healthcare provider may be either a man or a woman	
**5. Total time spent at facility (waiting and undertaking evaluation)**	2 hours	5 hours	8 hours
**6. Incentive for undertaking TB testing**	0 Kwacha	30 Kwacha (~$2USD)	60 Kwacha (~$4USD)
**7. TB test results (speed and notification method)**	TB testing results available before you leave	Contacted by phone with TB results and return instructions	Must return another day to facility for TB results

Prior to administering the discrete choice experiment, participants were asked to imagine that they were feeling sick again with their prior TB symptoms and they were then asked to choose at which one of the two facilities they would prefer to be evaluated. For each task, a participant was asked to select facility A or facility B based upon the different attribute levels shown, however, a participant could also select, "none, I wouldn’t choose either of these,” if neither hypothetical facility was acceptable.

The DCE was designed using Lighthouse Studio Version 9.7.2 (Sawtooth Software, Provo, USA). The final design was near balanced and orthogonal with respect to attributes and attribute levels [16]. Each participant was shown one of 400 randomly drawn choice sets comprised of 9 choice tasks [17]. Pictures accompanied all attributes and levels to enhance comprehension.

### DCE sample size estimation

The minimum sample size necessary for the DCE was determined in two ways. First, using the formula, “n ≥ 500c/ta,” where ‘n’ is the number of patients, ‘t’ is the number of choice tasks, ‘a’ is the number of options per choice task, and ‘c’ is the product of the greatest number of levels for any two attributes (when considering all two-way interactions) [12]; this resulted in a required sample size of n = 250 [(500 x 9)/(2x9)]. However, based upon the observation that there is limited improvement in estimate precision when a sample size of n = 300 is exceeded, a minimum of 300 participants is generally recommended if there are no planned subgroup analyses, or at least 200 participants per subgroup when subgroup analyses are planned [12,16,17]. As this study sought to evaluate differences in preferences according to HIV status, we aimed to recruit n = 400 (2*200) participants for inclusion.

### Definitions

‘Microbiologically-confirmed TB’ was defined by the detection of *Mycobacterium tuberculosis* using Xpert MTB/RIF or culture on any clinical specimen, and/or a positive sputum smear microscopy and/or urine Determine TB-LAM result. The ‘TB care pathway’ was defined as the time from self-reported symptom onset to initiation of TB therapy. ‘Health-seeking delay’ was defined as the time from symptom onset to initial care-seeking, ‘diagnostic delay’ was defined as the time from initial care-seeking to TB diagnosis, and ‘treatment delay’ was defined as the time from TB diagnosis to initiation of TB therapy.

### Analysis

We were *a priori* interested in evaluating whether patients’ TB care pathways, barriers and facilitators to TB care engagement and preferences differed according to HIV status. However, due to differences in baseline characteristics by enrollment site, analyses are presented both overall and according to HIV status by enrollment site. Proportions were compared using either Fisher’s exact tests or Pearson’s chi-squared tests as appropriate; median values were compared using Wilcoxon rank-sum tests. For the DCE, a hierarchical Bayesian (HB) model was used to estimate part-worth utilities as well as the relative importance of each facility’s attributes [12]. Sub-group analyses were undertaken to evaluate whether patient preferences differed according to HIV status, sex, or time to seeking care after symptom-onset (<4 weeks, ≥4 weeks) and were adjusted to account for differences by enrolment site. We then incorporated the calculated individual part-worth utilities into Sawtooth’s choice simulator tool to determine the additional distance participants would be willing to travel (in kilometers) or additional time they were willing to wait (in hours) in order to access enhanced healthcare facility features (attribute levels) [18].

## Results

### Baseline characteristics

**Fig 1:**
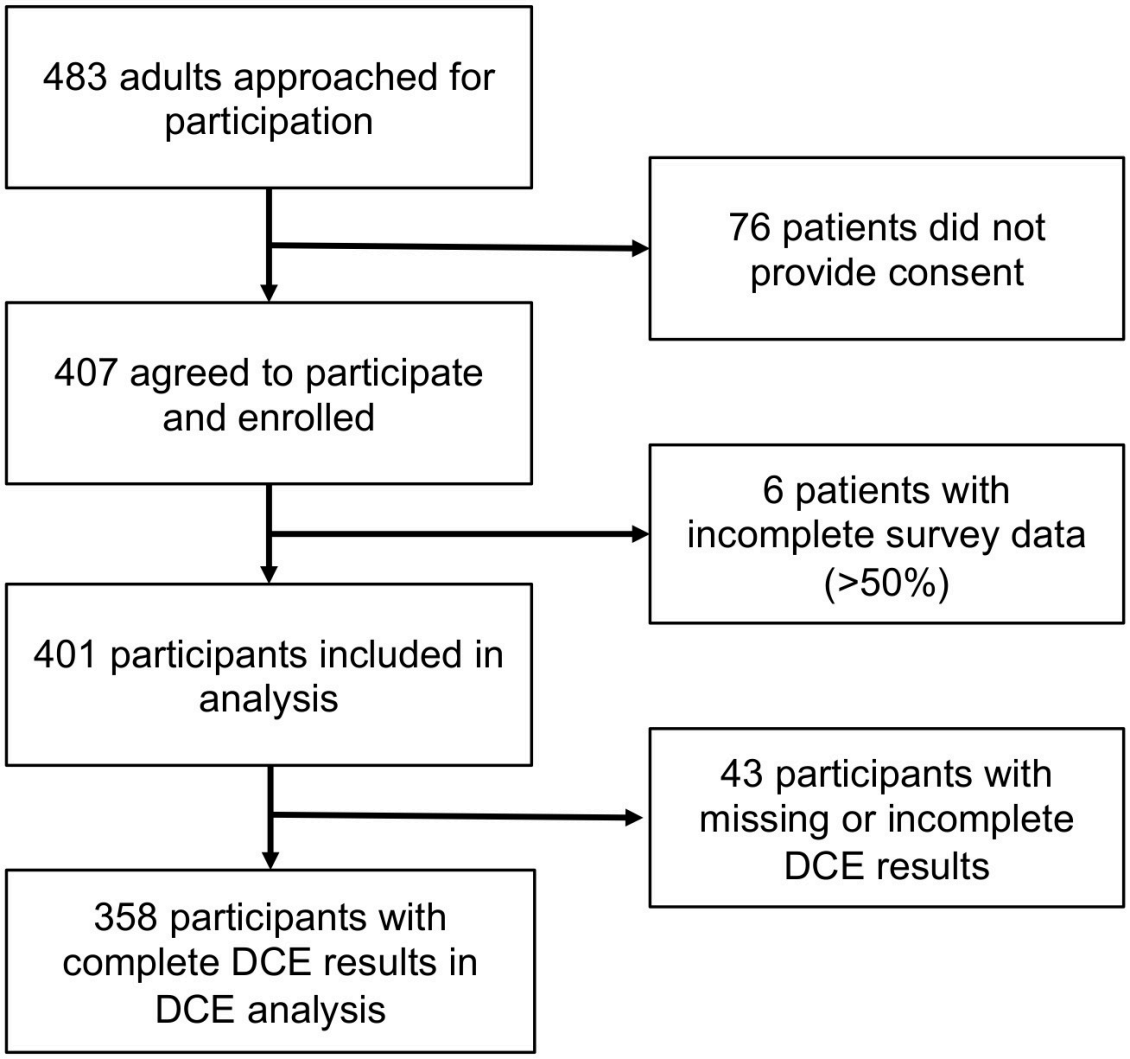
Study flow diagram.

Of 483 adults with newly confirmed TB recruited for study participation, 401 agreed to participate, had complete survey results, and were included (Fig 1); 60.8% were enrolled from the first level hospital, while 39.1% were enrolled from the tertiary hospital. TB patients tended to be young (median age 34 years), 68.7% were male, 46.6% were HIV-positive, and 14.3% had previously been treated for TB (Table 2).

**Table 2:** Baseline characteristics by enrollment site and HIV status.

	Overall	First-level Hospital		Tertiary Hospital	
	(n = 401)	HIV- positive (n = 78)	HIV- negative (n = 166)	HIV- positive (n = 109)	HIV- negative (n = 48)
**Age in years, median (IQR)**	34 (27–42)	35 (30–39)	30 (25–38)	36 (30–45)	33 (26–46)
**Sex**					
Male	275 (68.7)	46 (59.0)	145 (87.9)	53 (48.6)	31 (64.6)
Female	125 (31.3)	32 (41.0)	20 (12.1)	56 (51.4)	17 (35.4)
**Education**					
None/primary	172 (42.9)	41 (52.6)	77 (46.4)	41 (37.6)	13 (27.1)
Secondary/tertiary	229 (57.1)	37 (47.4)	89 (53.6)	68 (62.4)	35 (72.9)
**Relationship status**					
Currently married	192 (47.9)	43 (55.1)	73 (44.0)	59 (54.1)	17 (35.4)
Divorced or separated	49 (12.2)	20 (25.6)	20 (12.1)	5 (4.6)	4 (8.3)
Widowed	18 (4.5)	2 (2.6)	5 (3.0)	9 (8.3)	2 (4.1)
Unmarried	142 (35.4)	13 (16.7)	68 (41.0)	36 (33.0)	25 (52.1)
**Medical history**					
**HIV testing**					
Tested in last 12 months	273 (68.1)	23 (29.5)	161 (97.0)	46 (42.2)	43 (89.6)
Tested, but >12 months	124 (30.9)	55 (70.5)	5 (3.0)	61 (56.0)	3 (6.3)
Never tested	4 (1.0)	0	0	2 (1.8)	2 (4.2)
**If HIV-positive, ART status**					
Current daily use	172 (92.5)	70 (89.7)	-	102 (94.4)	-
Current use, occasional missed doses	5 (2.7)	3 (3.9)	-	2 (1.9)	-
Naïve	9 (4.8)	5 (6.4)	-	4 (3.7)	-
**If HIV-positive, attendance at ART clinic in last 3 months?**					
Yes	176 (95.1)	71 (91.0)	-	105 (97.2)	-
No	10 (5.4)	7 (9.0)	-	3 (2.8)	-
**Previous history of tuberculosis**					
Yes	57 (14.2)	13 (16.9)	14 (8.4)	23 (21.2)	7 (14.6)
No	344 (85.8)	65 (83.3)	152 (91.6)	86 (78.9)	41 (85.4)
**Monthly household income in Zambian Kwacha, median (IQR)**	1500 (900–3000)	1400 (900–3000)	1500 (900–3000)	1600 (1000–3800)	2000 (1000–4000)

Patient characteristics differed substantially by enrollment site and HIV status (Table 2). HIV-positive TB patients tended to be older and have a previous history of TB compared to HIV-negative TB patients, whilst the large majority of HIV-negative TB patients were men (Table 2). HIV-positive TB patients attending the tertiary hospital were more likely to have been diagnosed with HIV in the last 12 months than those attending the first-level facility (42.2% vs. 28.6%).

### Duration of TB care pathways

**Fig 2:**
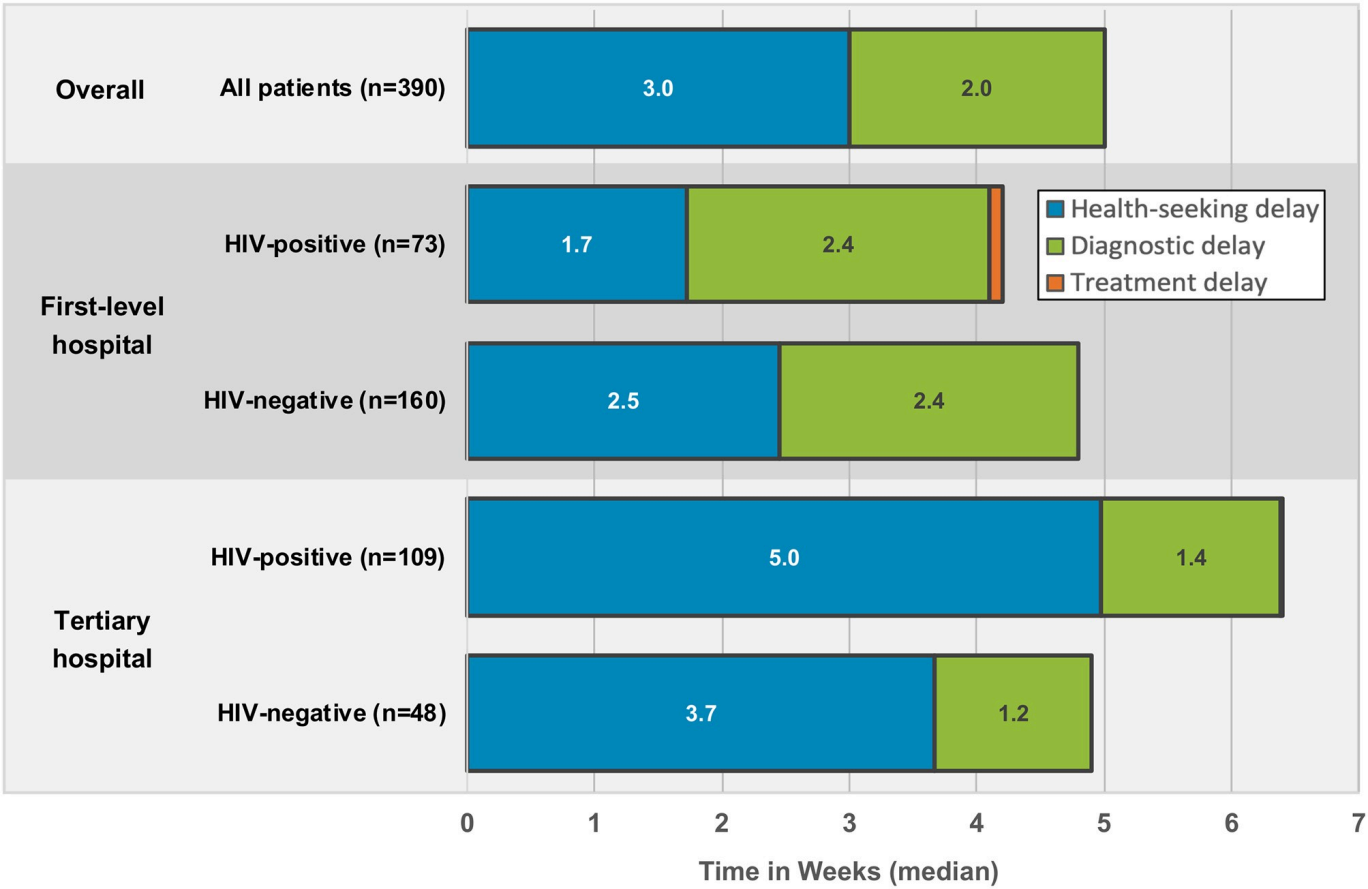
Tuberculosis patient care pathways in Zambia, according to HIV status by enrolment site.

The median duration of the TB care pathway among all patients was 5.0 (interquartile range [IQR], 3.6–8.0) weeks (Fig 2 and S1a Fig). While 71.0% of patients had a total TB care pathway of less than 8 weeks, 18.2% and 10.8% had a pathway lasting 8–11.9 weeks and ≥12 weeks, respectively (Table 3). This did not differ by HIV status among patients attending the first level hospital; however, among those attending the tertiary hospital, HIV-positive patients experienced a substantially longer period of time from symptom onset to initiation of TB therapy than HIV-negative patients (6.4 vs. 4.9 weeks, p = 0.002) (Table 3, Fig 2 and S1a Fig). HIV-positive patients attending the tertiary hospital also had longer pathways than HIV-positive patients seeking care at the first-level hospital (6.4 vs. 4.1 weeks, p = 0.012).

**Table 3:** Overview of TB patient care pathway according to enrollment site and HIV status.

	Overall	First-level Hospital			Tertiary Hospital		
		HIV- positive (n = 78)	HIV- negative (n = 166)	P-value	HIV- positive (n = 109)	HIV- negative (n = 48)	P-value
**Total TB pathway in weeks (median, IQR)**	5.0 (3.6–8.0)	4.1 (3.1–9.7)	4.9 (3.0–8.0)	0.58	6.4 (4.7–8.9)	4.9 (3.6–6.7)	0.002
**Total TB Pathway category**							
<4 weeks	119 (30.5)	27 (37.0)	63 (39.4)	0.21	15 (13.8)	14 (29.2)	0.06
4–7.9 weeks	158 (40.5)	23 (31.5)	52 (32.5)		57 (52.3)	26 (54.2)	
8–11.9 weeks	71 (18.2)	10 (13.7)	31 (19.4)		25 (22.9)	5 (10.4)	
≥12 weeks	42 (10.8)	13 (17.8)	14 (8.8)		12 (11.0)	3 (6.3)	
**Health-seeking delay in weeks (median, IQR)**	3.0 (1.0–5.0)	2.0 (1.0–3.0)	2.0 (1.0–4.0)	0.33	4.0 (3.0–6.0)	3.0 (2.0–5.0)	0.002
**Health-seeking delay category**							
<4 weeks	229 (58.7)	59 (80.8)	111 (69.4)	0.18	34 (31.2)	25 (52.1)	0.039
4–7.9 weeks	118 (30.3)	9 (12.3)	34 (21.3)		56 (51.4)	19 (39.6)	
≥8 weeks	43 (11.0)	5 (6.9)	15 (9.4)		19 (17.4)	4 (8.3)	
**Diagnostic delay in weeks (median, IQR)**	1.7 (0.9–3.0)	2.0 (0.9–6.3)	2.0 (1.0–3.9)	0.60	1.3 (0.9–2.4)	1.0 (0.9–2.0)	0.64
**Diagnostic delay category**							
<4 weeks	310 (79.5)	51 (69.9)	120 (75.0)	0.030	96 (88.1)	43 (89.6)	0.60
4–7.9 weeks	53 (13.6)	9 (12.3)	29 (18.1)		10 (9.1)	5 (10.4)	
≥8 weeks	27 (6.9)	13 (17.8)	11 (6.9)		3 (2.8)	0	
**Treatment delay in days (median, IQR)**	0 (0–0)	0 (0–1)	0 (0–0)	0.013	0 (0–0)	0 (0–0)	0.81
**Treatment delay category**							
<3 days	367 (91.5)	69 (88.5)	147 (88.6)	0.81	106 (97.3)	45 (93.8)	0.37
3–6 days	21 (5.2)	4 (5.1)	11 (6.6)		3 (2.8)	3 (6.3)	
≥7 days	13 (3.2)	5 (6.4)	8 (4.8)		0	0	

### Health-seeking delays (patient-related delays)

The majority of the time spent along the TB care pathway was due to patient-related delays in health-seeking (Fig 2 and S1b Fig). The overall median time from symptom onset to initial care-seeking was 3.0 weeks (IQR, 1.0–5.0). Most patients sought care for their TB illness within 4 weeks of symptom onset (58.7%), but 30.3% waited 4–7.9 weeks to first seek care, and 11.0% waited ≥8 weeks. Health-seeking delays differed by site and HIV status (Table 3, Fig 2 and S1b Fig). Prolonged delays were most common among HIV-positive patients attending the tertiary hospital—68.8% of such patients waited ≥4 weeks to seek care.

### Diagnostic and treatment delays (system-related delays)

The median diagnostic delay (time to diagnosis) after patients first sought care was 1.7 (IQR, 0.9–3.0) weeks (Fig 2 and S1c Fig). The large majority (79.5%) of patients were diagnosed with TB within 4 weeks of initially seeking care; however, 13.6% and 6.9% were diagnosed with TB 4–7.9 weeks and ≥8 weeks after first seeking care, respectively. Diagnostic delays were more common among patients attending the first-level hospital, especially HIV-positive patients (Table 3, Fig 2 and S1c Fig). Prolonged delays in initiation of TB therapy after establishing a TB diagnosis were uncommon (Table 3, Fig 2); 91.5% of patients were initiated on therapy within 2 days of diagnosis, whereas 5.2% were initiated on therapy 3–6 days after diagnosis, and 3.2% were initiated on therapy ≥7 days after diagnosis. Treatment delays did not differ by enrollment site or HIV status (Table 3).

### Facilities visited and treatments received during TB care pathway

Overall, patients visited a median of 1 (IQR 1–2) provider/facility prior to their TB diagnosis (S1 Table); however, 35.4% visited 2 providers/facilities and 8.2% made ≥3 different healthcare-related visits before their TB was diagnosed. When patients initially sought care for their illness, 86.8% attended a public health facility, 7.8% visited a pharmacy, 4.5% visited a private health facility and 1.0% sought care from a traditional healer. 75.6% of patients reported receipt of antibiotics during the course of their illness and 8.7% reported that they had received a traditional remedy. The number of facilities visited, location of initial care-seeking and treatments received did not substantially differ by enrollment site or HIV status (S1 Table).

### Factors influencing TB health-seeking behaviors

**Fig 3:**
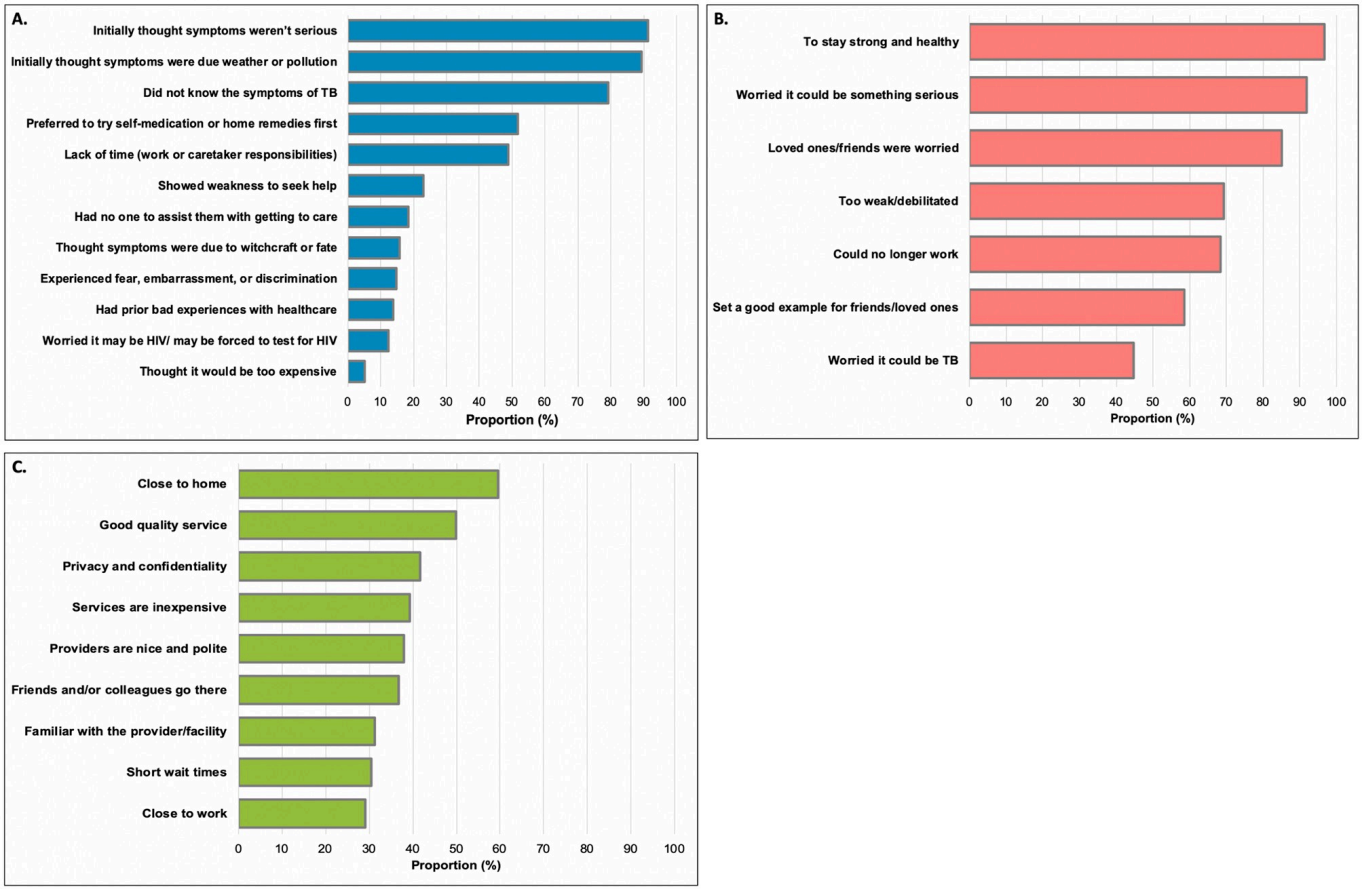
Barriers and facilitators to tuberculosis care engagement in Zambia: (a) reasons for delayed health-seeking among patients who contemplated presenting to care sooner than they did (n = 217); (b) reasons for seeking healthcare (n = 401); (c) reasons for choosing initial site/facility for evaluation and care (n = 401).

More than half (54.1%) of TB patients reported that they had contemplated presenting to care sooner than they did (S2 Table). Patients cited several reasons for delaying care-seeking, including: they initially thought their symptoms weren’t serious (91.2%), they thought that their symptoms were due to alternative causes such as weather or pollution (89.4%), they did not know the symptoms of TB (79.3%), they preferred to first try self-medication and/or home remedies (51.6%), and they lacked sufficient time due to work or caretaker responsibilities (48.9%) (Fig 3a). Less commonly reported reasons for delayed care-seeking included: it shows weakness, lack of social support, fear of discrimination/stigma and fear of being HIV-positive (Fig 3a). HIV-positive patients attending the first-level hospital were more likely to state that a fear of showing weakness influenced their delayed health-seeking (36.5 vs. 19.2%; p = 0.015), whilst HIV-negative patients were more likely to state that their concern about either having HIV or being required to test for HIV delayed initial care-seeking (16.7% vs. 3.9%; p = 0.021) (S2 Table).

Patients reported many factors influencing their decision to ultimately seek care for their illness, including: to stay healthy (96.8%), they feared it was something serious (91.8%), friends/loved ones were worried (85.0%), they were very weak and debilitated (69.3%), they could no longer work (68.5%), to set a good example for friends and loved ones (58.5%) and because they specifically feared that it could be TB (44.8%) (Fig 3b, S2 Table). TB patients chose an initial provider/facility for evaluation and treatment of their illness because it: was close to home (59.6%), offered good quality service (49.9%), offered privacy/confidentiality (41.7%), provided inexpensive services (39.2%), had friendly providers (37.9%), was a place their friends and/or loves ones went (36.7%), was a place they were familiar with (31.2%), and offered short wait times (30.4%) (Fig 3c, S2 Table).

### Patient preferences for improving TB services

**Fig 4:**
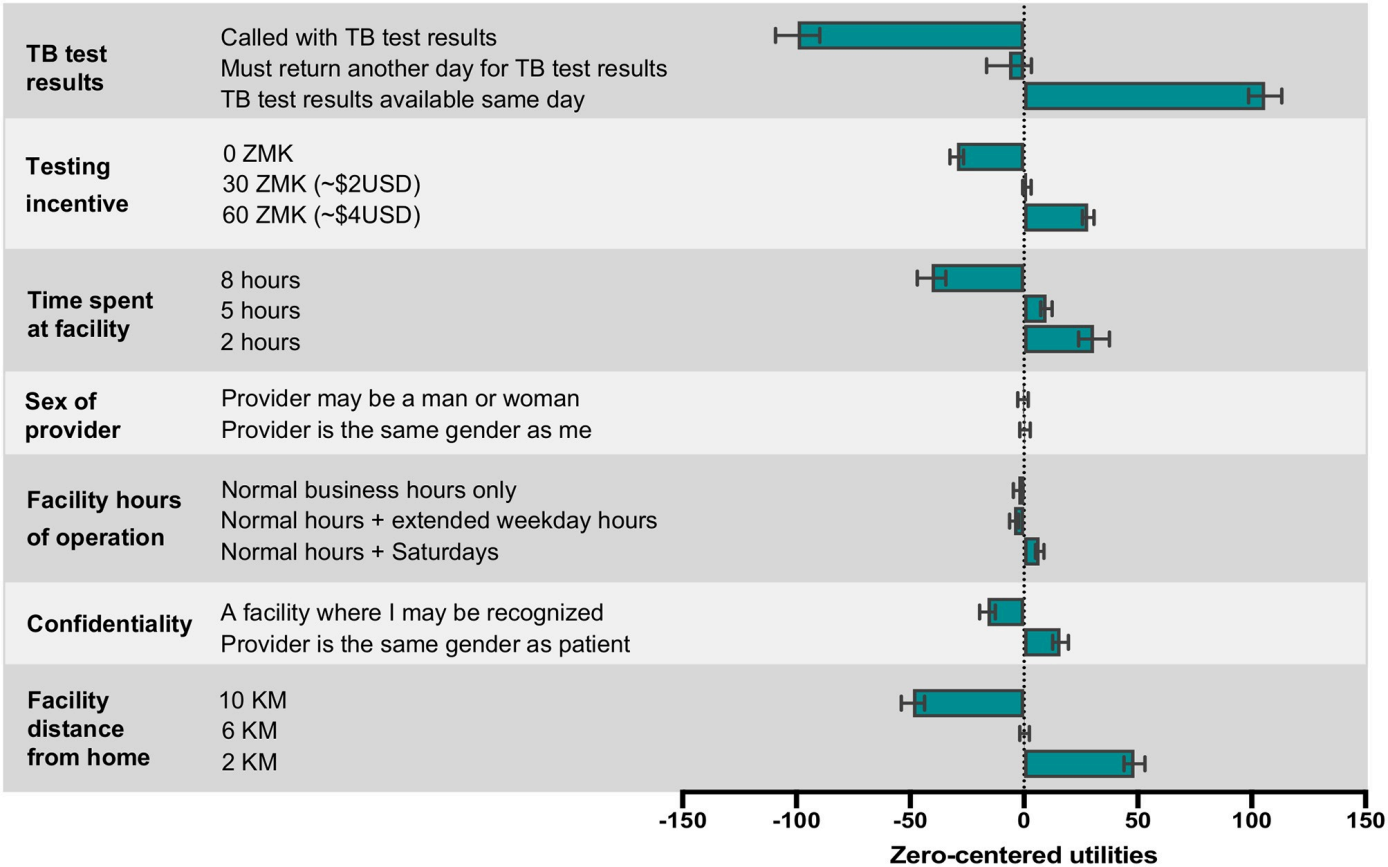
Patient preferences for improving tuberculosis care services in Zambia: The average utilities (zero-centered) for enhanced TB facility features. Abbreviations: KM = kilometers, TB = tuberculosis, ZMK = Zambian Kwacha.

Next, we determined patient preferences for improving TB diagnosis and care services. Patients very strongly preferred same-day TB test results compared to being contacted by phone with their results or returning another day to collect their results (Fig 4). They also showed strong preferences for shorter times spent at the facility, a facility closer to their home, a facility providing a financial incentive for undertaking TB testing and a facility that offered greater privacy/confidentiality. Patients demonstrated weak preferences for the facility hours of operation and the sex of their healthcare provider (Fig 4).

The most important service attribute was the speed/method of TB results (relative importance, 37.2%) (S2 Fig)—this was more than twice as important as the second and third most important attributes—the total amount of time spent at a facility (18.0%), and a facility’s proximity to their home (16.9%). In all sub-group analyses, the speed/method of TB results was the most important service attribute (range of relative importance across sub-groups, 31.0–39.6%) (S3–S5 Tables); the total time spent at a facility (range, 17.3–18.7%) and a facility’s proximity to home (range, 15.2–18.4%) also remained highly preferred attributes across all sub-group analyses.

**Fig 5:**
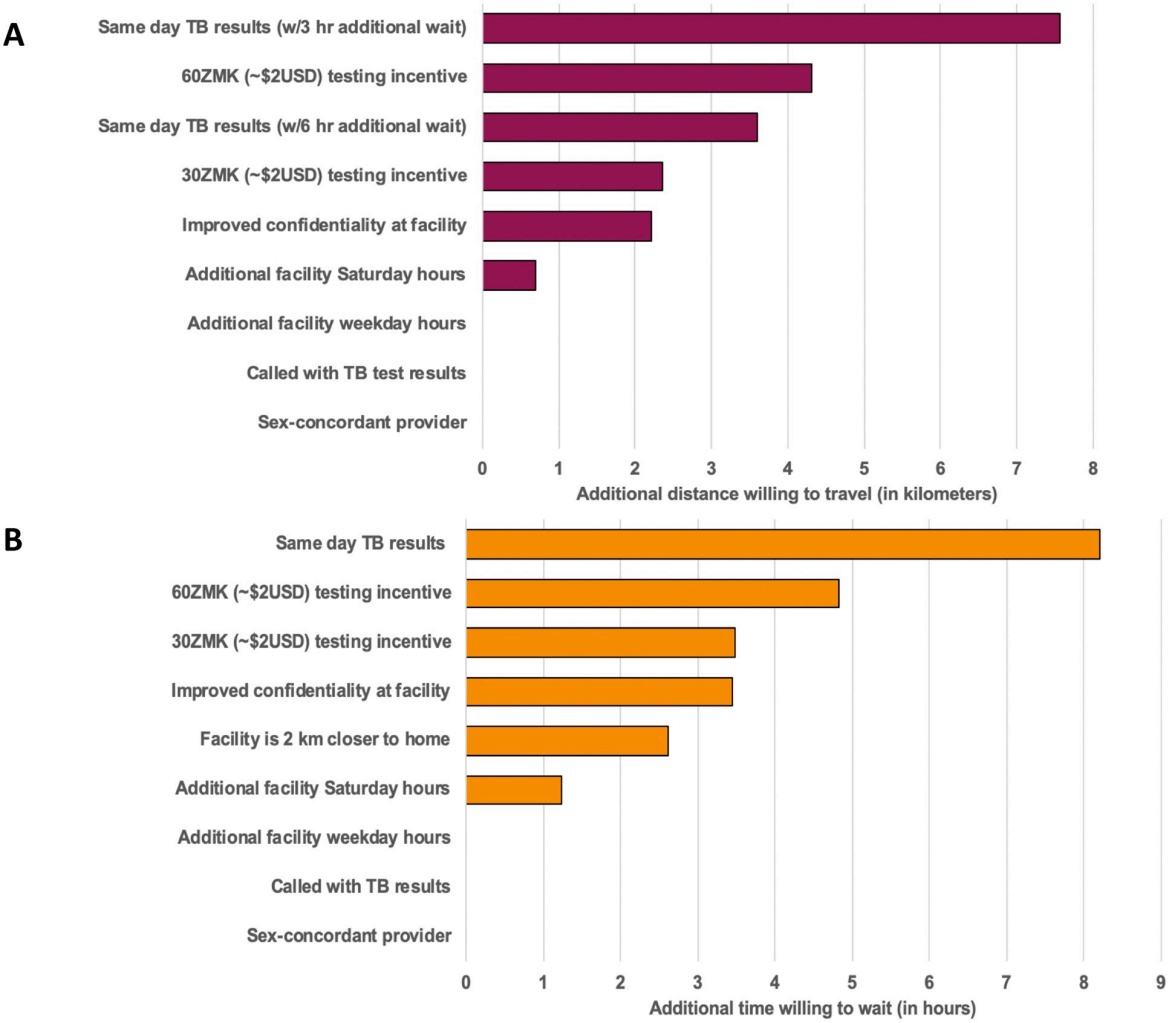
Tradeoffs patients were willing to make in order to access a facility offering different enhanced services: (a) additional willingness to travel (in kilometers), (b) additional willingness to wait (in hours). Willingness to travel and wait values were calculated using the following assumptions for a “usual” tuberculosis health facility in Lusaka, Zambia: it is 2 kilometers from an individual’s home, requires two hours spent at the clinic waiting and undergoing evaluation (based on the median amount of time cited by survey participants on their date of TB diagnosis), it is only be open during typical business hours Monday through Friday, an individual may be known or recognized there, is does not offer sex-concordant health care providers, it does not offer financial incentives for undergoing TB testing, and it requires individuals to return on a different day to collect their TB test results. Because it is not feasible to implement same-day TB testing without an additional wait time for patients, in the willingness’s to travel analyses, we assumed that patients would need to wait an additional 3 hours (minimum estimated time required for sample collection and transport, Xpert testing, and result notification), or 6 hours (total wait time of 5 or 8 hours, respectively), in order to access same-day TB testing.

Patients were willing to travel up to 7.6 kilometers further or wait an additional 8.2 hours in order to have access to a facility offering same-day TB test results (Fig 5). Patients were also willing to travel further and wait longer for a facility that offered financial incentives to undertake TB testing or a facility that provided enhanced confidentiality where they were unlikely to be known/recognized by anyone (Fig 5).

## Discussion

In this study among newly diagnosed TB patients in Lusaka, Zambia, we found that nearly 30% of patients had a TB care pathway that exceeded two months, and that prolonged care pathways were predominantly due to patient-related, health-seeking delays. More than half of patients said they had delayed health-seeking for which they reported several reasons and barriers, including a lack of TB-specific knowledge and a lack of time due to competing responsibilities. When asked about potential strategies to improve TB services that may facilitate earlier engagement, patients expressed very strong preferences for a facility providing same-day TB test results—they were willing to travel more than 7 kilometers further, or wait 8 hours longer to access this feature. These results have important implications for future strategies to improve TB diagnosis in high burden settings.

The median time from symptom onset to initiation of TB therapy among patients in our study was 5 weeks. A study also conducted in Lusaka two decades ago found that the TB care pathway was nearly 9 weeks [19]. While this suggests progress over the last 20 years, prolonged TB care pathways in excess of 2–3 months remained common in our study and were most frequently driven by patient-related delays in care-seeking. TB patients on average reported waiting 3 weeks before seeking care for their illness, but an important minority of 11% of patients waited 8 or more weeks. It is increasingly recognized that a small proportion of people with TB known as ‘superspreaders’ may contribute to the majority of community transmission through prolonged periods of infectiousness and a large number of contacts [20]. Thus, community-based, active TB case finding strategies to identify such patients sooner are likely to be an important component to improve TB control [21,22].

Several factors contributing to delayed healthcare-seeking behavior and TB diagnoses were identified. Many patients who delayed care-seeking stated that they either didn’t initially think their symptoms were serious and/or they didn’t know the symptoms of TB. This suggests the need for ongoing community-targeted awareness activities to disseminate TB information and education, including the individual and societal benefit of early TB diagnosis and treatment as well as where to go for evaluation [23,24]. While less commonly reported by study participants, a perception that it shows weakness to get help, fear of discrimination after TB diagnosis, and a lack of social support contribute to TB health-seeking delays in this setting; additionally, 15–30% of HIV-negative patients said they delayed care-seeking, in part because of a fear of having HIV. This highlights the need to normalize healthy behaviors, prevent and mitigate TB- and HIV-related stigma and improve social support as part of multicomponent strategies to facilitate earlier TB care engagement [25,26].

In addition to frequent and prolonged delays in health-seeking, once patients did seek care, more than 40% visited two or more providers/facilities before being diagnosed with TB—further prolonging the TB care pathway. Most of such patients (90%) first visited a clinic or hospital suggesting that their TB diagnosis was missed in at least one healthcare encounter. In high prevalence settings, systematic TB screening of all individuals presenting to care for any reason, may yield a large number of TB cases that may have otherwise been missed [27–30]. In settings where this may not be feasible, facilities and TB control programs should focus on prioritizing systematic TB screening among persons with a high risk of TB, including, PLHIV, persons with diabetes, close contacts of TB patients and individuals with a prior history of TB treatment [30,31]. Implementation strategies to facilitate broader uptake of this should be evaluated. Prompt initiation of TB treatment following diagnosis was a programmatic strength of facilities included in our study as 92% of patients started TB treatment within two days of diagnosis.

PLHIV attending a large, tertiary referral hospital had substantially longer TB care pathways compared to HIV-negative TB patients attending the same facility as well as PLHIV attending a first-level hospital. These differences were largely accounted for by delays in initial health-seeking. The reasons provided for delaying care-seeking did not differ according to HIV status and there were also no differences in HIV care engagement services by enrolment site. Thus, it is not clear why such patients were more likely to delay their care-seeking. PLHIV attending the tertiary hospital may have been more likely to have advanced immunodeficiency resulting in non-specific symptoms that were initially overlooked and/or caused them to wait because they were not felt be serious; however, we were unable evaluate this possibility as CD4 counts were not systematically determined. To reduce preventable suffering and deaths due to TB among PLHIV, further scale-up of collaborative TB and HIV services are needed [32,33].

Among potential strategies to improve TB services and accelerate care engagement, TB patients expressed very strong preferences for same-day TB test results. The availability of same-day TB results was more than twice as preferred as the next most preferred improvement strategy (total time spent at a facility) and was the most preferred TB service attribute, regardless of HIV status, sex, or the time to initial care-seeking. We also found that patients would be willing to travel substantially further (more than 7 additional kilometers) or wait substantially longer (more than 8 additional hours) in order to access a health facility providing same-day TB test results. In addition to being a convenient, patient-centered strategy that could facilitate earlier care-seeking, same-day TB testing and treatment initiation reduces diagnostic and treatment delays as well as pre-treatment loss-to-follow-up [34,35]; it may also reduce prolonged periods of infectiousness among those with untreated TB [35]. Xpert/Xpert Ultra is currently recommended as a first-line TB diagnostic test due to its substantially improved sensitivity over smear microscopy and its ability to simultaneously detect rifampicin resistance [36]; however, due to cost and infrastructure requirements, Xpert testing machines are often not available on site, especially at lower healthcare facility levels, and results are therefore rarely available the same-day [37]. Development and implementation of rapid, accurate, point-of-care triage tests [31,38] and confirmatory tests [39,40] among presumptive TB patients in high-burden settings could facilitate same-day rule-out or confirmation of TB, respectively.

TB patients also voiced strong preferences for shorter wait times and facilities close to their home. In addition to same-day test results, these attributes each relate to a reduction in time spent seeking care and evaluation and imply the value that socioeconomically vulnerable individuals place on their time. Therefore, additional strategies that could facilitate earlier TB detection may include decentralization of TB services to community clinics (e.g., health posts as part of a hub and spoke model) as well as TB fast-track points at health facilities where close contacts and individuals with TB symptoms can skip the regular que and undergo rapid screening and testing for TB [28].

Strengths of this study include the inclusion of a large number of consecutive, newly diagnosed TB patients from two different levels of healthcare facilities. There are however some study limitations. Health-seeking delays were based upon patients’ self-report and may be subject to either recall or desirability bias. While patients’ TB care pathways and TB service preferences are likely representative of individuals in Lusaka Province, which accounts for at least 40% of Zambia’s TB cases [41], individuals from additional regions in Zambia, including rural settings were not included. Finally, although we intentionally included patients presenting to a tertiary hospital as a proxy for very delayed care-seeking, this study does not account for patients who never sought care for their TB illness.

In conclusion, among newly diagnosed TB patients in a high TB/HIV setting, prolonged TB care pathways were common and were predominantly driven by patients’ delays in health-seeking after symptom onset. Among potential strategies to facilitate earlier TB care engagement, patients strongly preferred TB facilities that offered same-day TB test results. To detect more TB cases and identify them sooner, future interventions must address local barriers to care engagement, and should directly incorporate patients’ preferences, especially same-day TB test results.

## Supporting information

S1 Fig Violin plots of the tuberculosis patient care pathways in Zambia, according to HIV status by enrolment site: (a) total patient pathway (in weeks); (b) health-seeking delay (in weeks); (c) diagnostic delay (in weeks). The bold dashed line represents the 50^th^ percentile value (median), while the fine dashed lines represent the 25^th^ and 75^th^ percentile values.
(TIF)

S2 Fig Patient preferences for improving tuberculosis care services in Zambia: The relative importance of TB facility attributes. (TIF)

S1 Table Overview of facilities visited and interventions received as part of the TB care pathway, according to HIV-status by enrolment site. (DOCX)

S2 Table Barriers and facilitators to tuberculosis care engagement. (DOCX)

S3 Table Average zero-centered utility values for TB service attributes and their relative importance according to HIV-status. (DOCX)

S4 Table Average zero-centered utility values for TB service attributes and their relative importance according to sex. (DOCX)

S5 Table Average zero-centered utility values for TB service attributes and their relative importance according to self-reported time duration from onset of TB symptoms to initial care-seeking for evaluation (in weeks). (DOCX)

S1 Appendix Structured survey. (DOCX)
