# Supplementary figures and images for: Pathways to care and preferences for improving tuberculosis services among tuberculosis patients in Zambia: A discrete choice experiment
Source: PLoS One. 2021 Aug 31;16(8):e0252095. doi: 10.1371/journal.pone.0252095 (PMC8407587; doi:10.1371/journal.pone.0252095)

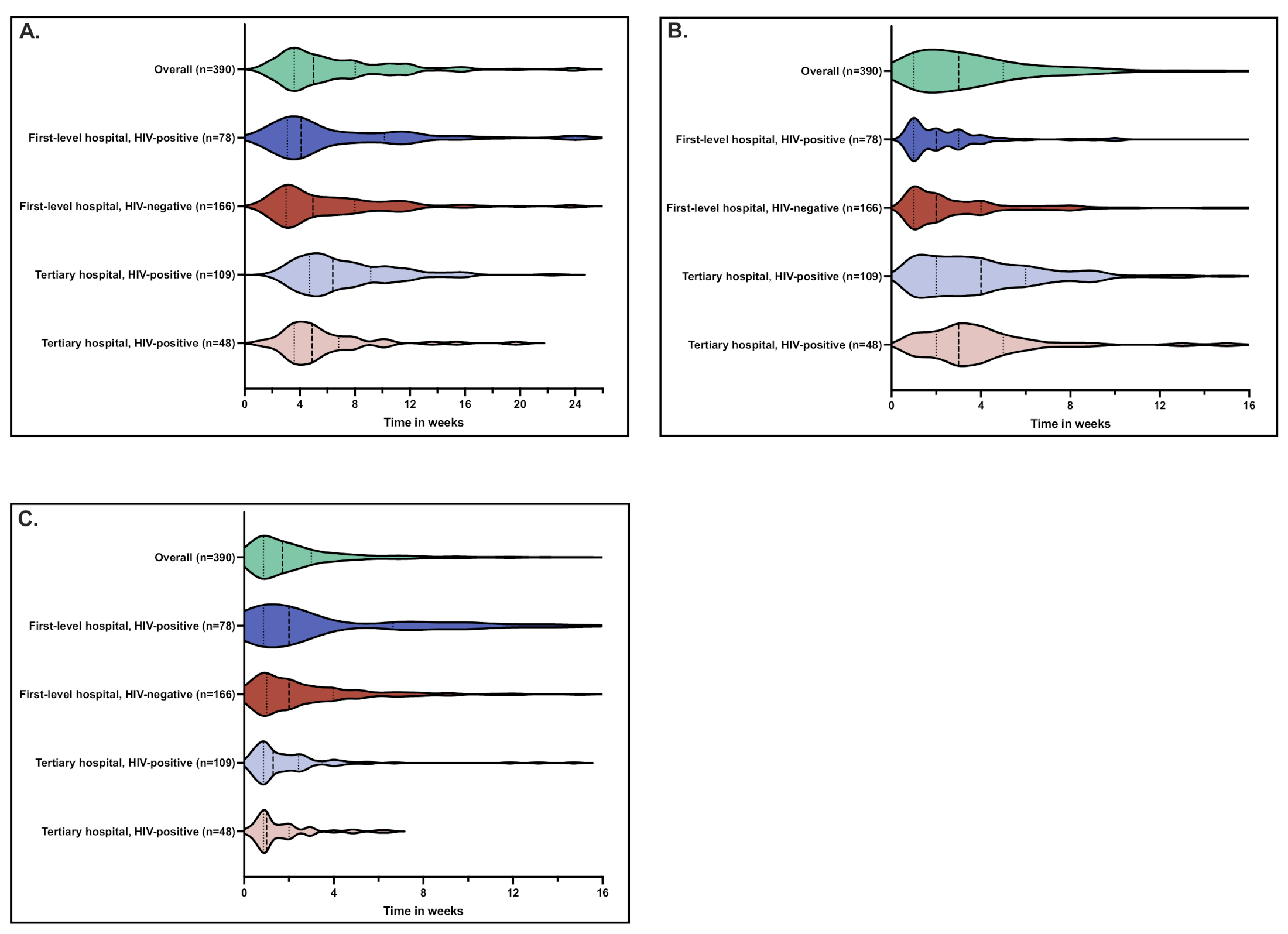

Supplement: S1 Fig — The bold dashed line represents the 50th percentile value (median), while the fine dashed lines represent the 25th and 75th percentile values. (TIF) [file pone.0252095.s001.tif]

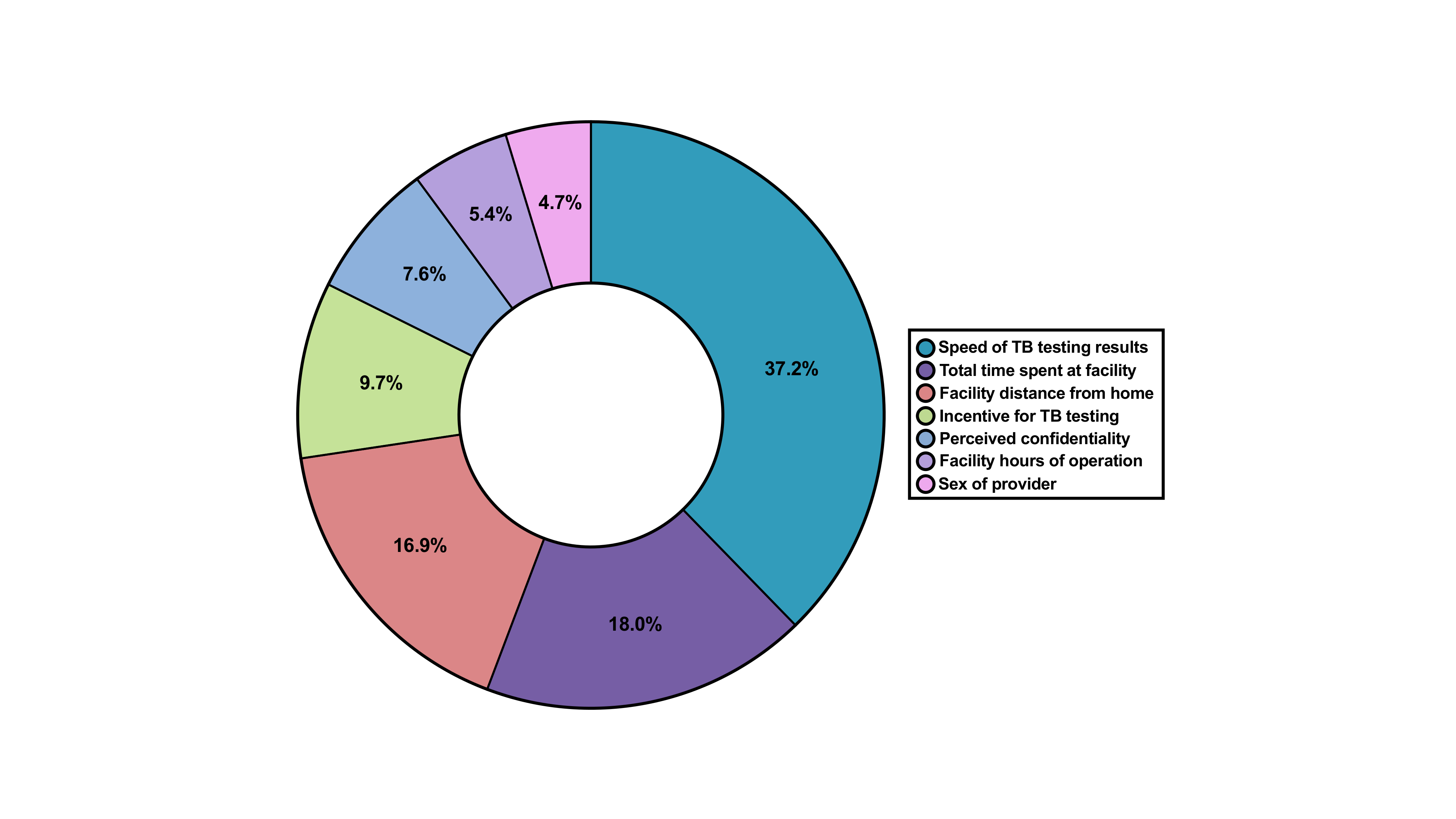

Supplement: S2 Fig — (TIF) [file pone.0252095.s002.tif]
