# Supplementary material for: Pathways to care and preferences for improving tuberculosis services among tuberculosis patients in Zambia: A discrete choice experiment
Source: PLoS One. 2021 Aug 31;16(8):e0252095. doi: 10.1371/journal.pone.0252095 (PMC8407587; doi:10.1371/journal.pone.0252095)
Supplement: S1 Table — (DOCX) [file pone.0252095.s003.docx]

**S1 Table. Overview of facilities visited and interventions received as part of the TB care pathway, according to HIV-status by enrolment site.**

|  | **Overall** | **First-level Hospital** | | | **Tertiary Hospital** | | | |
| --- | --- | --- | --- | --- | --- | --- | --- | --- |
|  |  | **HIV-**  **Positive**  **(n=78)** | **HIV-Negative**  **(n=166)** | **P-value** | | **HIV-Positive**  **(n=109)** | **HIV-Negative**  **(n=48)** | **P-value** |
| **Number of facilities visited, (median, IQR)** | 1 (1-2) | 1 (1-2) | 1 (1-2) | 0.48 | | 1 (1-2) | 1 (1-2) | 0.78 |
| **Number of facilities visited, total** |  |  |  |  | |  |  |  |
| 1 | 226 (56.4) | 41 (52.6) | 98 (59.0) | 0.43 | | 61 (56.0) | 26 (54.2) | 0.88 |
| 2 | 142 (35.4) | 30 (38.5) | 50 (30.1) |  | | 43 (39.5) | 19 (36.6) |  |
| ≥3 | 33 (8.2) | 7 (9.0) | 18 (10.8) |  | | 5 (4.6) | 3 (6.3) |  |
| **Site first visited by patients for TB illness** |  |  |  |  | |  |  |  |
| Public clinic or hospital | 347 (86.8) | 64 (82.1) | 136 (81.9) | 0.011 | | 102 (93.6) | 45 (93.8) | 1 |
| Private clinic or hospital | 18 (4.5) | 1 (1.3) | 8 (4.8) |  | | 6 (5.5) | 3 (6.3) |  |
| Pharmacy | 31 (7.8) | 8 (10.3) | 22 (13.3) |  | | 1 (0.9) | 0 |  |
| Herbalist/traditional healer | 4 (1.0) | 4 (5.2) | 0 |  | | 0 | 0 |  |
| Other | 1 (0.3) | 1 (1.3) | 0 |  | | 0 | 0 |  |
| **Therapies received prior to TB diagnosis** |  |  |  |  | |  |  |  |
| Traditional remedy | 35 (8.7) | 9 (11.5) | 14 (8.4) | 0.44 | | 7 (6.4) | 5 (10.4) | 0.39 |
| Antibiotics | 303 (75.6) | 58 (74.4) | 134 (80.7) | 0.26 | | 79 (72.5) | 32 (66.7) | 0.46 |
