# Supplementary material for: Pathways to care and preferences for improving tuberculosis services among tuberculosis patients in Zambia: A discrete choice experiment
Source: PLoS One. 2021 Aug 31;16(8):e0252095. doi: 10.1371/journal.pone.0252095 (PMC8407587; doi:10.1371/journal.pone.0252095)
Supplement: S2 Table — (DOCX) [file pone.0252095.s004.docx]

**S2 Table. Barriers and facilitators to tuberculosis care engagement.**

|  | **Overall**  **(n=401)** | **First-level Hospital** | | | **Tertiary Hospital** | | |
| --- | --- | --- | --- | --- | --- | --- | --- |
|  |  | **HIV-Positive**  **(n=78)** | **HIV-Negative**  **(n=166)** | **P-**  **value** | **HIV-Positive**  **(n=109)** | **HIV-Negative**  **(n=48)** | **P-**  **value** |
| **Contemplated presented to care sooner than they did** | 217 (54.1) | 52 (66.7) | 120 (72.3) | 0.37 | 35 (32.1) | 10 (20.8) | 0.15 |
| **Reasons cited for delayed care-seeking (n=217)** |  |  |  |  |  |  |  |
| Thought symptoms weren’t serious | 198 (91.2) | 49 (94.2) | 110 (91.7) | 0.76 | 30 (85.7) | 9 (90.0) | 1 |
| Thought symptoms were due to other causes (pollution, weather) | 194 (89.4) | 45 (86.5) | 112 (93.3) | 0.15 | 27 (77.1) | 10 (100) | 0.17 |
| Did not know the symptoms of TB | 172 (79.3) | 40 (76.9) | 98 (81.7) | 0.47 | 26 (74.3) | 8 (80.0) | 0.71 |
| Preferred to try self-medication or home remedies first | 112 (51.6) | 22 (42.3) | 49 (40.8) | 0.86 | 33 (94.3) | 8 (80.0) | 0.21 |
| Lack of time (work or caretaker responsibilities) | 106 (48.9) | 31 (59.6) | 56 (46.7) | 0.12 | 13 (37.1) | 6 (60.0) | 0.28 |
| Showed weakness to seek help | 50 (23.0) | 19 (36.5) | 23 (19.2) | 0.015 | 7 (20.0) | 1 (10.0) | 0.47 |
| Had no one to assist them with getting to care | 40 (18.4) | 7 (13.5) | 26 (21.7) | 0.21 | 6 (17.1) | 1 (10.0) | 1 |
| Thought symptoms were due to witchcraft or fate | 34 (15.7) | 7 (13.5) | 23 (19.2) | 0.37 | 4 (11.4) | 0 | 0.56 |
| Experienced fear, embarrassment, or discrimination | 32 (14.8) | 5 (9.6) | 20 (16.7) | 0.35 | 7 (20.0) | 0 | 0.32 |
| Had prior bad experiences with healthcare | 30 (13.8) | 4 (7.7) | 13 (10.8) | 0.53 | 10 (28.6) | 3 (30.0) | 1 |
| Worried it may be HIV/ may be forced to test for HIV | 27 (12.4) | 2 (3.9) | 20 (16.7) | 0.021 | 2 (5.7) | 3 (30.0) | 0.07 |
| Thought it would be too expensive | 11 (5.1) | 1 (1.9) | 5 (4.2) | 0.67 | 4 (11.4) | 1 (10.0) | 0.90 |
| **Reasons for seeking care** |  |  |  |  |  |  |  |
| To stay strong and healthy | 387 (96.8) | 76 (97.4) | 163 (98.2) | 0.66 | 104 (95.4) | 45 (93.8) | 0.70 |
| Worried it could be something serious | 367 (91.8) | 72 (92.3) | 159 (95.8) | 0.26 | 99 (90.8) | 38 (79.2) | 0.044 |
| Loved ones/friends were worried | 340 (85.0) | 69 (88.5) | 147 (88.6) | 0.98 | 87 (79.8) | 38 (79.2) | 0.93 |
| Too weak/debilitated | 277 (69.3) | 66 (84.6) | 123 (74.1) | 0.07 | 63 (57.8) | 25 (52.1) | 0.51 |
| Could no longer work | 274 (68.5) | 60 (76.9) | 119 (71.7) | 0.39 | 70 (64.2) | 25 (52.1) | 0.15 |
| Set a good example for loved ones | 234 (58.5) | 29 (37.2) | 82 (49.4) | 0.07 | 91 (83.5) | 33 (68.8) | 0.37 |
| Worried it could be TB | 179 (44.8) | 27 (34.6) | 54 (32.5) | 0.75 | 70 (64.2) | 19 (60.4) | 0.65 |
| **Reasons for choosing first site/facility for care** |  |  |  |  |  |  |  |
| Close to home | 239 (59.6) | 56 (71.8) | 121 (72.9) | 0.86 | 41 (37.6) | 21 (43.8) | 0.47 |
| Good quality service | 200 (49.9) | 21 (26.9) | 52 (31.3) | 0.48 | 91 (83.5) | 36 (75.0) | 0.21 |
| Privacy and confidentiality | 167 (41.7) | 14 (18.0) | 38 (22.9) | 0.38 | 82 (75.2) | 33 (68.8) | 0.40 |
| Services are inexpensive | 157 (39.2) | 17 (21.8) | 44 (26.5) | 0.43 | 67 (61.5) | 29 (60.4) | 0.90 |
| Providers are nice and polite | 152 (37.9) | 16 (19.2) | 38 (22.9) | 0.52 | 72 (66.1) | 27 (56.3) | 0.24 |
| Friends and/or colleagues go there | 147 (36.7) | 27 (34.6) | 57 (34.3) | 0.97 | 45 (41.3) | 18 (37.5) | 0.66 |
| Familiar with the provider/facility | 125 (31.2) | 26 (33.3) | 44 (26.5) | 0.27 | 38 (34.9) | 17 (35.4) | 0.94 |
| Short wait times | 122 (30.4) | 21 (26.9) | 46 (27.2) | 0.90 | 46 (42.2) | 9 (18.8) | 0.005 |
| Close to work | 116 (29.0) | 30 (38.5) | 60 (36.1) | 0.73 | 18 (16.5) | 8 (16.7) | 0.98 |
