# Supplementary material for: Pathways to care and preferences for improving tuberculosis services among tuberculosis patients in Zambia: A discrete choice experiment
Source: PLoS One. 2021 Aug 31;16(8):e0252095. doi: 10.1371/journal.pone.0252095 (PMC8407587; doi:10.1371/journal.pone.0252095)
Supplement: S3 Table — (DOCX) [file pone.0252095.s005.docx]

**S3 Table. Average zero-centered utility values for TB service attributes and their relative importance according to HIV-status.**

|  | **HIV-positive**  **(n=170)** | **HIV-negative**  **(n=187)** |
| --- | --- | --- |
| **Facility distance from home** |  |  |
| 2 KM | 40.2 (32.2, 48.2) | 49.8 (44.4, 55.2) |
| 6 KM | -0.4 (-4.3, 3.5) | -0.7 (-4.3, 3.0) |
| 10 KM | -39.8 (-47.8, -31.8) | -49.1 (-56.4, -41.9) |
| **Confidentiality** |  |  |
| A place where no one knows who I am | 14.7 (9.4, 19.9) | 15.9 (10.9, 20.9) |
| A place where I may be known or recognized | -14.7 (-19.9, -9.4) | -15.9 (-20.9, -10.9) |
| **Facility hours of operation** |  |  |
| Normal weekday hours | -4.4 (-7.8, -0.9) | -0.5 (-3.7, 2.6) |
| Normal weekday hours and extra morning and evening hours | -5.8 (-8.8, -2.9) | -3.5 (-6.5, -0.4) |
| Normal weekday hours and open on Saturdays | 10.2 (7.1, 13.3) | 4.0 (0.9, 7.0) |
| **Sex-concordance of provider** |  |  |
| The healthcare provider is the same sex as me | 4.4 (0.6, 8.1) | -5.2 (-8.7, -1.8) |
| The healthcare provider may be a man or a woman | -4.4 (-8.1, -0.6) | 5.2 (1.8, 8.7) |
| **Total time spent at facility (waiting and evaluation)** |  |  |
| 2 hours | 34.0 (24.7, 43.3) | 25.5 (16.3, 34.6) |
| 5 hours | 19.4 (15.9, 23.0) | 2.7 (-1.8, 7.1) |
| 8 hours | -53.5 (-62.3, -44.6) | -28.1 (-36.9, -19.3) |
| **Incentive for TB testing and (value in Kwacha)** |  |  |
| 0 Kwacha ($0 USD) | -30.0 (-34.4, -25.6) | -25.7 (-30.0, -21.3) |
| 30 Kwacha (~$2 USD) | 0.8 (-2.2, 3.9) | 0.3 (-2.5, 3.1) |
| 60 Kwacha (~$4 USD) | 29.2 (24.6, 33.8) | 25.4 (22.1, 28.7) |
| **TB testing results** |  |  |
| TB testing results available before you leave (same-day) | 101.1 (89.6, 112.5) | 99.5 (91.5, 107.5) |
| Contacted by phone with TB testing results and return instructions | -68.8 (-81.7, -55.9) | -118.8 (-132.5, -105.2) |
| Must return to clinic another day to collect results | -32.3 (-46.5, -18.1) | 19.3 (6.5, 32.2) |
| **Relative importance of facility features** |  |  |
| Total distance from home | 16.6 (15.1, 18.0) | 16.5 (15.1, 17.9) |
| Perceived confidentiality | 8.0 (6.9, 9.1) | 7.3 (6.2, 8.5) |
| Facility hours of operation | 6.4 (5.8, 7.0) | 6.2 (5.6, 6.7) |
| Sex-concordant provider | 5.4 (4.6, 6.1) | 5.1 (4.4, 5.8) |
| Total time spent at facility (waiting and evaluation) | 18.7 (17.0, 20.3) | 17.3 (15.9, 18.7) |
| Incentive for testing and result collection (value in Kwacha) | 10.6 (9.6, 11.5) | 9.5 (8.8, 10.3) |
| Speed and notification of TB test results | 34.5 (32.0, 36.9) | 38.1 (36.1, 40.1) |

*Model was adjusted for enrolment site
