# Supplementary material for: Pathways to care and preferences for improving tuberculosis services among tuberculosis patients in Zambia: A discrete choice experiment
Source: PLoS One. 2021 Aug 31;16(8):e0252095. doi: 10.1371/journal.pone.0252095 (PMC8407587; doi:10.1371/journal.pone.0252095)
Supplement: S4 Table — (DOCX) [file pone.0252095.s006.docx]

**S4 Table. Average zero-centered utility values for TB service attributes and their relative importance according to sex.**

|  | **Men**  **(n=242)** | **Women**  **(n=115)** |
| --- | --- | --- |
| **Facility distance from home** |  |  |
| 2 KM | 43.7 (37.3, 50.0) | 45.8 (38.7, 52.8) |
| 6 KM | 3.7 (1.2, 6.3) | -4.5 (-10.5, 1.5) |
| 10 KM | -47.4 (-54.0, -40.7) | -41.2 (-51.1, -31.4) |
| **Confidentiality** |  |  |
| A place where no one knows who I am | 16.6 (12.7, 20.5) | 13.3 (5.9, 20.6) |
| A place where I may be known or recognized | -16.6 (-20.5, -12.7) | -13.3 (-20.6, -5.9) |
| **Facility hours of operation** |  |  |
| Normal weekday hours | -3.3 (-6.2, -0.4) | -0.2 (-4.8, 4.4) |
| Normal weekday hours and extra morning and evening hours | -0.7 (-3.1, 1.7) | -12.1 (-17.2, -7.0) |
| Normal weekday hours and open on Saturdays | 4.0 (1.4, 6.6) | 12.3 (7.2, 17.4) |
| **Sex-concordance of provider** |  |  |
| The healthcare provider is the same sex as me | -1.0 (-3.6, 1.7) | 0.7 (-4.1, 5.4) |
| The healthcare provider may be a man or a woman | 1.0 (1.4, 6.6) | -0.7 (-5.4, 4.1) |
| **Total time spent at facility (waiting and evaluation)** |  |  |
| 2 hours | 24.7 (16.4, 32.9) | 39.9 (29.7, 50.2) |
| 5 hours | 9.4 (6.0, 12.7) | 15.3 (11.0, 19.6) |
| 8 hours | -34.0 (-42.3, -25.8) | -55.2 (-64.7, -45.8) |
| **Incentive for TB testing and (value in Kwacha)** |  |  |
| 0 Kwacha ($0 USD) | -22.5 (-26.0, -19.1) | -40.9 (-47.6, -34.1) |
| 30 Kwacha (~$2 USD) | -1.2 (-3.8, 1.4) | 5.6 (2.0, 9.2) |
| 60 Kwacha (~$4 USD) | 23.8 (20.8, 26.7) | 35.3 (28.9, 41.6) |
| **TB testing results** |  |  |
| TB testing results available before you leave (same-day) | 98.6 (89.8, 107.4) | 103.3 (92.1, 114.4) |
| Contacted by phone with TB testing results and return instructions | -113.3 (-126.4, -100.2) | -62.5 (-75.3, -49.6) |
| Must return to clinic another day to collect results | 14.7 (2.6, 26.7) | -40.8 (-54.9, -26.7) |
| **Relative importance of facility features** |  |  |
| Total distance from home | 16.9 (15.7, 18.2) | 16.0 (14.3, 17.7) |
| Perceived confidentiality | 7.3 (6.5, 8.2) | 8.6 (7.1, 10.2) |
| Facility hours of operation | 5.5 (5.0, 6.1) | 8.9 (8.2, 9.6) |
| Sex-concordant provider | 4.3 (3.7, 4.8) | 5.6 (4.7, 6.5) |
| Total time spent at facility (waiting and evaluation) | 18.1 (16.8, 19.3) | 17.4 (15.2, 19.5) |
| Incentive for testing and result collection (value in Kwacha) | 8.9 (8.2, 9.5) | 12.6 (11.1, 14.1) |
| Speed and notification of TB test results | 39.0 (37.0, 41.0) | 31.0 (28.4, 33.6) |

*Model was adjusted for enrolment site

**Supplementary Table 6.** Average zero-centered utility values for TB service attributes and their relative importance according to self-reported time duration from onset of TB symptoms to initial care-seeking for evaluation (in weeks)

|  | **<4 weeks**  **(n=200)** | **≥4 weeks**  **(n=147)** |
| --- | --- | --- |
| **Facility distance from home** |  |  |
| 2 KM | 47.4 (42.4, 52.5) | 43.4 (34.2, 52.6) |
| 6 KM | -5.8 (-9.1, -2.5) | 6.1 (1.9, 10.2) |
| 10 KM | -41.6 (-48.6, -34.6) | -49.5 (-58.7, -40.2) |
| **Confidentiality** |  |  |
| A place where no one knows who I am | 8.8 (4.9, 12.6) | 24.0 (17.7, 30.3) |
| A place where I may be known or recognized | -8.8 (-12.6, -4.9) | -24.0 (-30.3, -17.7) |
| **Facility hours of operation** |  |  |
| Normal weekday hours | -6.1 (-8.6, -3.5) | 1.9 (-2.5, 6.4) |
| Normal weekday hours and extra morning and evening hours | -11.2 (-13.7, -8.8) | 4.6 (1.0, 8.3) |
| Normal weekday hours and open on Saturdays | 17.3 (14.9, 19.7) | -6.6 (-10.6, -2.5) |
| **Sex-concordance of provider** |  |  |
| The healthcare provider is the same sex as me | -0.9 (-3.8, 1.9) | 0.2 (-4.2, 4.6) |
| The healthcare provider may be a man or a woman | 0.9 (-1.9, 3.8) | -0.2 (-4.6, 4.2) |
| **Total time spent at facility (waiting and evaluation)** |  |  |
| 2 hours | 24.8 (15.3, 34.4) | 31.7 (22.3, 41.1) |
| 5 hours | 13.3 (9.2-17.4) | 9.2 (5.1, 13.4) |
| 8 hours | -38.2 (-46.6, -29.7) | -40.9 (-51.8, -30.0) |
| **Incentive for TB testing and (value in Kwacha)** |  |  |
| 0 Kwacha ($0 USD) | -24.3 (15.3, 34.4) | -31.4 (-35.4, -27.5) |
| 30 Kwacha (~$2 USD) | -3.8 (9.2, 17.4) | 6.0 (1.9, 10.0) |
| 60 Kwacha (~$4 USD) | 28.1 (-46.6, -29.7) | 25.5 (20.5, 30.5) |
| **TB testing results** |  |  |
| TB testing results available before you leave (same-day) | 93.6 (84.0-103.1) | 105.3 (95.0-115.7) |
| Contacted by phone with TB testing results and return instructions | -121.6 (-136.9, -106.4) | -59.4 (-68.9, -50.0) |
| Must return to clinic another day to collect results | 28.1 (14.9-41.3) | -45.9 (-58.6, -33.2) |
| **Relative importance of facility features** |  |  |
| Total distance from home | 15.2 (13.8, 16.6) | 18.4 (16.6, 20.1) |
| Perceived confidentiality | 5.8 (5.0, 6.6) | 9.3 (7.8, 10.8) |
| Facility hours of operation | 6.4 (5.9, 6.9) | 7.1 (6.4, 7.9) |
| Sex-concordant provider | 4.6 (4.0, 5.1) | 5.5 (4.6, 6.4) |
| Total time spent at facility (waiting and evaluation) | 18.3 (16.8, 19.7) | 17.8 (16.0, 19.5) |
| Incentive for testing and result collection (value in Kwacha) | 10.2 (9.4, 11.1) | 10.6 (9.6, 11.5) |
| Speed and notification of TB test results | 39.6 (37.2, 41.9) | 31.4 (29.4, 33.5) |

*Model was adjusted for enrolment site
