# Supplementary material for: Pathways to care and preferences for improving tuberculosis services among tuberculosis patients in Zambia: A discrete choice experiment
Source: PLoS One. 2021 Aug 31;16(8):e0252095. doi: 10.1371/journal.pone.0252095 (PMC8407587; doi:10.1371/journal.pone.0252095)
Supplement: S1 Appendix — (DOCX) [file pone.0252095.s008.docx]

**S1 Appendix. Structured survey.**

| **Question number** | **Variable Name** | | **Question** | **Variable type and responses** |
| --- | --- | --- | --- | --- |
| **Section 1: Identification and study screen (To be completed by interviewer)** | | | | |
|  | STUDYDATE | | Date of interview | Free Form |
|  | STUDYID | | Study Participant ID | Free Form |
|  | STUDYID2 | | To ensure data integrity, please re-enter the Study participant ID. | Free Form |
|  | INTNUM | | Interviewer number | Numeric |
| **Section 2: Patient basic demographics** | | | | |
|  | LANGUAGE | | What is your preferred language? (choose only one) | Select:   1. Bemba 2. Nyanja 3. English |
|  | Date of Birth | | What is your date of birth? (enter 01/01/XX if patient only knows the year they were born) | Free Form (date) (Skip to 4 if patient knows their DOB) |
|  | Age | | What is your age? (in years) (SKIPPED if DOB completed above) | Numeric (in years) |
|  | GENDER | | What is your gender? | Select:   1. Male 2. Female |
|  | EDUCATION | | What is your highest level of education started? | Select:   1. Never attended school 2. Primary school 3. Secondary school 4. Any tertiary (University or College) |
|  | RELATIONSHIP | | What is your marital status? | Select:   1. Currently married 2. Divorced or separated 3. Widowed 4. Single |
|  | FAITH | | Do you consider yourself to be religious or is faith important to you? | Select:   1. Yes, I go to church every week or almost every week 2. Yes, but I don’t often go to church 3. No |
|  | ADDRESS | | What landmark is your house number closest to? (store, gas station, etc) | Free Form |
|  | TIMECLINIC | | How long did it take you to get to this facility? | Select:   1. <10 minutes 2. 10-30 minutes 3. 30-60 minutes 4. 1-2 hours 5. >2 hours |
|  | TRANSPORT | | How did you get to this facility? | Select:   1. Walk 2. Bicycle 3. Motorbike 4. Private car/taxi 5. Minibus 6. Other |
|  | MINIBUS | | How frequently do use a minibus for transportation? | Select:   1. Daily 2. 3 or more times a week 3. 1-2 times per week 4. A few times a month 5. Never |
| **Section 3: Medical and social history** | | | | |
|  | PASTTB | Have you ever been treated for TB disease in the past? | | Select:   1. Yes 2. No |
|  | KNOWTB | Has anyone you know been diagnosed with TB? | | Select:   1. Yes, in the last 6 months 2. Yes, but not in the last 6 months 3. No (Skip to 15) |
|  | TBCONTACT | If you said that you knew someone who has been diagnosed with TB in the last 6 months, what is their relationship to you? | | Select:  1. Friend  2. Family member  3. I know a friend AND a family member who have been diagnosed with TB in the last 6 months. |
|  | TBSUSPECTED | Do you know anyone that you think may have TB, but has not yet been diagnosed with TB (or been told that they have TB)? | | 1. Yes, a friend or colleague  2. Yes, a family member  3. Yes, both at least one friend/colleague and at least one family member  4. No |
|  | HIVTEST | Have you ever been tested for HIV? | | Select:   1. Yes, in last 12 months 2. Yes, more than 12 months ago 3. Never been tested 4. Not sure |
|  | HIVRESULT | Is your HIV status positive? | | Select:   1. Yes, positive 2. No, negative (Skip to 20) 3. Not sure |
|  | ART | If you are HIV-positive, are you taking ART? | | Select:   1. Yes, take everyday 2. Yes, last dose within 7 days 3. No, previously on ART but stopped 4. Never been on ART |
|  | HIVCARE | If you are HIV-positive, have you been seen in the HIV clinic in the last 3 months? | | Select:   1. Yes 2. No |
|  | DM | Have you ever been diagnosed with or told that you have diabetes (high blood sugar)? | | Select:   1. Yes 2. No |
|  | HTN | Have you ever been diagnosed with or told that you have hypertension (high blood pressure)? | | Select:   1. Yes 2. No |
|  | SMOKEEVER | In the past, have you smoked tobacco on a daily basis, less than daily or not at all? | | Select:   1. Daily 2. Less than daily 3. Not at all |
|  | CURRENTSMOKER | Do you currently smoke tobacco on a daily basis, less than daily, or not at all? | | Select:   1. Daily 2. Less than daily 3. Not at all |
|  | ALCOHOL-AUDITC1 | Before you became ill, how often did you have a drink containing alcohol? | | Select:   1. Never (Skip to 27) 2. Monthly or less 3. 2-4 times a month 4. 2-3 times a week 5. 4 or more times a week |
|  | ALCOHOL-AUDITC2 | Before your became ill, how many drinks containing alcohol do you have on a typical day when you are drinking? | | 1. 0 2. 1 or 2 3. 3 or 4 4. 5 or 6 5. 7, 8, or 9 6. 10 or more |
|  | ALCOHOL-AUDITC3 | Before your became ill, were there times when you have six or more drinks containing alcohol? | | 1. Never 2. Less than monthly 3. Monthly 4. Weekly 5. Daily or almost daily |
|  | BAR | How frequently do you go to a bar, pub or night club? | | Select:   1. Daily 2. 3 or more times a week 3. 1-2 times per week 4. A few times a month 5. Never |
| **Section 4: Patient economic characteristics** | | | | |
|  | EMPLOYMENT | | Are you currently employed or have a way to generate money? | Select   1. Yes, office job 2. Yes, casual work (non-office work, gardener, house) 3. Yes, piecework (odd jobs here and there) 4. Yes, self-employed 5. No, currently a student 6. No, stopped working when I became ill 7. No, I don’t work 8. Other (please explain) _____ |
|  | INCOMEGEN | | Do you provide all/or the majority of the income for your household? | Select:   1. Yes 2. No |
|  | DAILYINCOME | | Approximately how much money do you make in a day? (if multiple jobs, all jobs combined) (amount in Kwacha) | Free form (in ZMK) |
|  | HOUSEHOLDINCOME | | What is the approximate combined income of your family each month? (amount in Kwacha) | Free form (amount in ZMK) |
| **Section 5: Patient pathways and delays** | | | | |
|  | SYMPTOMONSET | | Looking back, when do you think you first started having symptoms for this episode of TB? (in reference to current date- i.e., how many weeks ago?) If patient isn’t sure about symptoms – interviewer should then ask, “when do you think you first started experiencing cough, weight loss, fevers, night sweats or chest pain?” (number of weeks) | Free form (number of weeks) |
|  | OTHERSYMPTOMS | | What symptoms have you experienced? (Interviewer- check all that patient mentioned*), interviewer should ask about each symptom below and record either yes or no.* | Select:   1. Cough (2 or more weeks, less than 2 weeks, none) 2. Chest pain 3. Sputum production 4. Fevers 5. Night sweats 6. Accidental weight loss 7. Shortness of breath 8. Weakness 9. Fatigue 10. Loss of appetite 11. Body pains 12. Other_____(specify) |
|  | FIRSTPROVIDER | | Now, I would like you to name all the persons, clinics or hospitals that you visited for care or advice for symptoms of your current illness (including hospitalizations)?  Where did you go first? | Select:   1. This facility 2. Other public clinic 3. Private clinic 4. Public hospital 5. Private hospital 6. Pharmacy / Drugstore 7. Herbalist / traditional practitioners 8. Community Health Worker 9. Other |
|  | DELAYWEEKS | | How many weeks ago did you visit the 1^st^ provider for symptoms of your current illness? | Free form (weeks) |
|  | SEEKINGDELAY | | How many weeks after your symptoms started, did you wait to see the 1^st^ provider? | Free form (weeks) |
|  | FIRSTPROVIDERTESTS | | Which of the following, did you undertake or receive at this visit (1^st^ provider)? (Interviewer- check all that apply) | Select:   1. Chest X-ray 2. Blood test 3. Provided sputum sample 4. Antibiotic prescription 5. Traditional herbs/remedies |
|  | WHYFIRSTPROVIDER | | Why did you choose to go to this provider first? (Interviewer, read all possibilities and check all that apply, if other record reason listed by patient) | Select:   1. Close to home 2. Close to work 3. Short wait times 4. Services are inexpensive 5. Good quality service 6. Privacy/confidentiality 7. Providers are nice/polite 8. Familiar with the provider (seen them before) 9. My friends and colleagues go there 10. Other (describe) – open form |
|  | SECONDPROVIDER | | Where did you visit 2^nd^? (provider type) | Select:   1. N/A (skip to 49) 2. This facility 3. Other public clinic 4. Private clinic 5. Public hospital 6. Private hospital 7. Pharmacy / Drugstore 8. Herbalist / traditional practitioners 9. Community Health Worker 10. Other |
|  | SECONDPROVIDERTESTS | | Which of the following, did you undertake or receive at this visit? (Interviewer- check all that apply)) | Select:   1. Chest X-ray 2. Blood test 3. Provided sputum sample 4. Antibiotic prescription 5. Traditional herbs/remedies |
|  | THIRDPROVIDER | | Where did you visit 3rd? (provider type) | Select:   1. N/A (skip to 49) 2. This facility 3. Other public clinic 4. Private clinic 5. Public hospital 6. Private hospital 7. Pharmacy / Drugstore 8. Herbalist / traditional practitioners 9. Community Health Worker 10. Other |
|  | THIRDPROVIDERTESTS | | Which of the following, did you undertake or receive at this visit? (Interviewer- check all that apply) | Select:   1. Chest X-ray 2. Blood test 3. Provided sputum sample 4. Antibiotic prescription 5. Traditional herbs/remedies |
|  | FOURTHPROVIDER | | Where did you visit 4th? (provider type) | Select:   1. N/A (skip to 49) 2. This facility 3. Other public clinic 4. Private clinic 5. Public hospital 6. Private hospital 7. Pharmacy / Drugstore 8. Herbalist / traditional practitioners 9. Community Health Worker 10. Other |
|  | FOURTHPROVIDERTESTS | | Which of the following, did you undertake or receive at this visit? (Interviewer- check all that apply) | Select:   1. Chest X-ray 2. Blood test 3. Provided sputum sample 4. Antibiotic prescription 5. Traditional herbs/remedies |
|  | FIFTHPROVIDER | | Where did you visit 5th? (provider type) | Select:   1. N/A (skip to 49) 2. This facility 3. Other public clinic 4. Private clinic 5. Public hospital 6. Private hospital 7. Pharmacy / Drugstore 8. Herbalist / traditional practitioners 9. Community Health Worker 10. Other |
|  | FIFTHPROVIDERTESTS | | Which of the following, did you undertake or receive at this visit? (Interviewer- check all that apply) | Select:   1. Chest X-ray 2. Blood test 3. Provided sputum sample 4. Antibiotic prescription 5. Traditional herbs/remedies |
|  | SIXTHPROVIDER | | Where did you visit 6th? (provider type) | Select:   1. N/A (skip to 49) 2. This facility 3. Other public clinic 4. Private clinic 5. Public hospital 6. Private hospital 7. Pharmacy / Drugstore 8. Herbalist / traditional practitioners 9. Community Health Worker 10. Other |
|  | SIXTHPROVIDERTESTS | | Which of the following, did you undertake or receive at this visit? (Interviewer- check all that apply) | Select:   1. Chest X-ray 2. Blood test 3. Provided sputum sample 4. Antibiotic prescription 5. Traditional herbs/remedies |
|  | TIMEWAITING | | On the day you that you presented to this facility and were diagnosed with TB, how long did you spend at the clinic that day (in hours - to the nearest hour)? This is calculated from the time that you first arrived at the clinic to the time you left. (Deactivated if patient enrolled at UTH) | Free form (hours) |
| **Section 6: Healthcare seeking behavior** | | | | |
|  | REASONDELAY | | After your symptoms started, did you consider seeking care/or evaluation earlier than you did? **If, yes why did you wait?** (Interviewer, read all possibilities slowly and check all that apply; if ‘other’ please type in the patient’s response.’) | Select:   1. No, did not consider presenting sooner/did not delay (Skip to 52) 2. Thought symptoms were not serious and would get better on their own 3. Thought symptoms were due to other causes (pollution, weather) 4. Thought symptoms were due to witchcraft, curse or fate 5. Preferred to try self-medication or home remedies first 6. Lack of time 7. Too expensive 8. It shows weakness to get/need help. 9. Fear, embarrassment, or discrimination 10. Worried it may be HIV or may be forced to test for HIV 11. I had no one to assist me 12. My family opposed it 13. Previous bad experience with healthcare 14. My religion prohibited it. 15. I did not know the symptoms of TB |
|  | DELAYTIME | | If you said that you did not present for care immediately after your symptoms started in part due to lack of time, which of these reasons contributed? (Interviewer- read all possibilities slowly and check all that apply) | Select:   1. N/A 2. Conflicts with work – loss of income that myself and/or my family. 3. May lose job which gives me pride and confidence. 4. Conflicts with caretaking – children or loved ones 5. Too many responsibilities (not related to work or caretaking) 6. Other (describe) – open form |
|  | WHYPRESENT? | | When you did present for your symptoms, what reasons made you want to be evaluated? (Interviewer, read all possibilities slowly and check all that apply, if ‘other’ please type in the patient’s response.’) | Select:   1. I wanted to stay strong and healthy 2. I was worried it could be something serious 3. I was worried it could be tuberculosis 4. I could no longer work 5. I was weak/debilitated 6. My loved ones/friends were worried/encouraged me to come 7. To set a good example for my loved ones 8. Other (describe) – open form |
|  | WHYNOTCLINIC | | Is this the first facility you presented to? If you did not first present to this current health facility for your symptoms, why not (check all that apply)? (Interviewer, if answer is no, please continue to read all possibilities and check all that apply; if ‘other’ please type in the patient’s response.’) | 1. Yes, 2. No, Long wait times 3. No, Rude providers 4. No, Facility is dirty 5. No, Facility is far away 6. No, Not private or confidential 7. No, Was not familiar with this clinic 8. No, I thought services were expensive 9. No, Facility is not for men (if male) women (if female) 10. No, my friends or colleagues would not come here 11. No, Other (describe) – open form |
|  | TBINFOREACH | | What are the sources of information that you think can most effectively reach people like you with information on TB (Interviewer, read all possibilities and ask the patient to choose all that apply; if ‘other’ please type in the patient’s response.’) | Select:   1. Newspapers and 2. magazines 3. Radio 4. TV 5. Billboards 6. Social media (internet) 7. Brochures, posters and other printed materials 8. Health workers 9. Family 10. Friends and coworkers 11. Neighbors 12. Religious leaders 13. Teachers 14. Plays in the community 15. 14. Other (describe) – open form |
| 55. | HEALTHINFLUENCE | | What types of people most influence decisions related to your health? (Interviewer, read all possibilities and ask the patient to choose all that apply; if ‘other’ please type in the patient’s response.’) | Select:   1. Nobody, only myself 2. Spouse/partner 3. Other family member 4. Friends 5. Coworkers 6. Neighbors 7. Religious leaders 8. Doctors / healthcare workers 9. Other (describe) – open form |
| **Section 7: Satisfaction** | | | | |
| 56. | OVERALLSATISFACTION | | Were you satisfied with the services you have received at this facility? | Select:   1. Yes, Satisfied 2. No, Dissatisfied 3. Not sure |
| 57. | MEETSNEEDS | | I feel like this facility addresses my specific needs as a man/woman (Interviewer, if patient is male, then state man, if patient is female, state woman) | Select:   1. Yes, Agree 2. No, Disagree 3. Not sure |
| 58. | GENDERPROVIDER | | What gender would you prefer that your healthcare provider be? | 1. Male 2. Female 3. It doesn’t matter to me |
| 59. | RECOMMENDLOVE | | How likely would you be to recommend this facility to a friend or loved one? | Select:   1. Likely 2. Unlikely 3. Not sure |
| **END OF SURVEY** | | | | |
